# Long-Term Effect of Ultraviolet Irradiation on Poly(vinyl chloride) Films Containing Naproxen Diorganotin(IV) Complexes

**DOI:** 10.3390/molecules24132396

**Published:** 2019-06-28

**Authors:** Angham G. Hadi, Emad Yousif, Gamal A. El-Hiti, Dina S. Ahmed, Khudheyer Jawad, Mohammad Hayal Alotaibi, Hassan Hashim

**Affiliations:** 1 Department of Chemistry, College of Science, Babylon University, Babil 51002, Iraq; 2 Department of Chemistry, College of Science, Al-Nahrain University, Baghdad 64021, Iraq; 3 Department of Optometry, College of Applied Medical Sciences, King Saud University, P.O. Box 10219, Riyadh 11433, Saudi Arabia; 4 Department of Medical Instrumentation Engineering, Al-Mansour University College, Baghdad 64021, Iraq; 5 National Center for Petrochemicals Technology, King Abdulaziz City for Science and Technology, P.O. Box 6086, Riyadh 11442, Saudi Arabia; 6 Department of Physics, College of Science, Al-Nahrain University, Baghdad 64021, Iraq

**Keywords:** naproxen, poly(vinyl chloride) films, diorganotin(IV) complexes, photodegradation, photostabilizer, synthesis

## Abstract

As poly(vinyl chloride) (PVC) photodegrades with long-term exposure to ultraviolet radiation, it is desirable to develop methods that enhance the photostability of PVC. In this study, new aromatic-rich diorganotin(IV) complexes were tested as photostabilizers in PVC films. The diorganotin(IV) complexes were synthesized in 79–86% yields by reacting excess naproxen with tin(IV) chlorides. PVC films containing 0.5 wt % diorganotin(IV) complexes were irradiated with ultraviolet light for up to 300 h, and changes within the films were monitored using the weight loss and the formation of specific functional groups (hydroxyl, carbonyl, and polyene). In addition, changes in the surface morphologies of the films were investigated. The diorganotin(IV) complexes enhanced the photostability of PVC, as the weight loss and surface roughness were much lower in the films with additives than in the blank film. Notably, the dimethyltin(IV) complex was the most efficient photostabilizer. The polymeric film containing this complex exhibited a morphology of regularly distributed hexagonal pores, with a honeycomb-like structure—possibly due to cross-linking and interactions between the additive and the polymeric chains. Various mechanisms, including direct absorption of ultraviolet irradiation, radical or hydrogen chloride scavenging, and polymer chain coordination, could explain how the diorganotin(IV) complexes stabilize PVC against photodegradation.

## 1. Introduction

Poly(vinyl chloride) (PVC) is one of the most important thermoplastic polymers produced on an industrial scale [1]. PVC can be produced either as a rigid material that is stiff and has a high resistance to chemicals, water, and weather or as a flexible material that is soft and has a low degree of crystallinity [2]. PVC is durable, has good mechanical and chemical properties, has low production costs, and is as a good insulator [3]. Owing to its high chlorine content, PVC can be used as a flame retardant [3]. In addition, it can be used in window frames, indoor plumbing, shower curtains, cable insulation, tubing, automobiles, and electronics [4,5]. However, PVC has low heat stability, with changes in shape and color occurring at high temperatures. Furthermore, long exposure of PVC to ultraviolet (UV) radiation, e.g., from sunlight, and a high temperature results in undesirable changes in its physical and chemical properties [6,7]. Therefore, considerable attention has been paid to stabilizing PVC against irradiation to reduce its photodegradation rate [8,9,10,11].

PVC photodegradation occurs mainly through an autocatalytic dehydrochlorination process. This process has reasonably high activation energy that can be achieved under sunlight at a high temperature. In the initiation step of the photodegradation process, structural defects form within PVC [12]. In the propagation step, the thermal degradation process leads to the formation of polyene residues owing to the elimination of hydrogen chloride (HCl) and the formation of double-bond-containing chains [13,14,15]. Interactions between the polyene chains lead to a cross-linked PVC structure [16]. Various additives, such as impact modifiers, functional agents, UV stabilizers, biocides, and antistatic agents, can be added to PVC to allow its use for certain applications [17,18,19]. In addition, other additives, such as Schiff basses [20,21,22,23], inorganic salts [24,25,26,27], aromatics [28,29], pigments [30], and flame retardants [30], can be used to protect PVC against irradiation.

We have previously shown that various aromatic-rich Sn(IV) complexes can be used as PVC photostabilizers [31,32,33]. In addition, such complexes can be used as carbon dioxide storage media owing to their high surface areas [34]. As a continuation of our work in the field of polymers [35,36,37,38,39,40,41], new Sn(IV) complexes containing naproxen, which is highly aromatic, were synthesized and their use as efficient photostabilizers to enhance the stability of PVC against UV irradiation were investigated. The addition of a low concentration (0.5 wt %) of these new Sn(IV) complexes to PVC films increased the photostability against long-term UV irradiation significantly.

## 2. Results and Discussion

### 2.1. Synthesis of Sn(IV) Complexes ***1**–**3***

**Figure 1:**
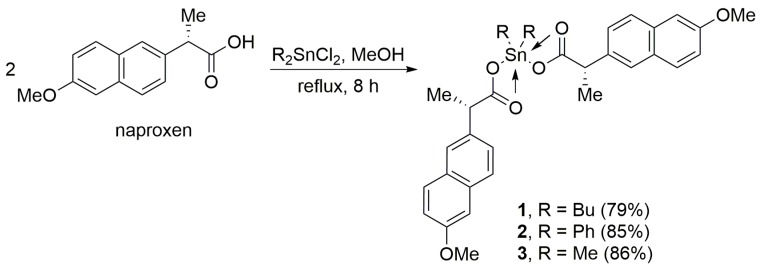
Synthesis of Sn(IV) complexes **1**–**3**.

Three naproxen Sn(IV) complexes (**1**–**3**) were synthesized in high yields (79–86%) via the reaction of excess naproxen (2 mol equivalents) and diorganotin(IV) chlorides (Figure 1). The physical properties and elemental analysis of Sn(IV) complexes **1**–**3** are reported in Table 1.

**Figure 2:**
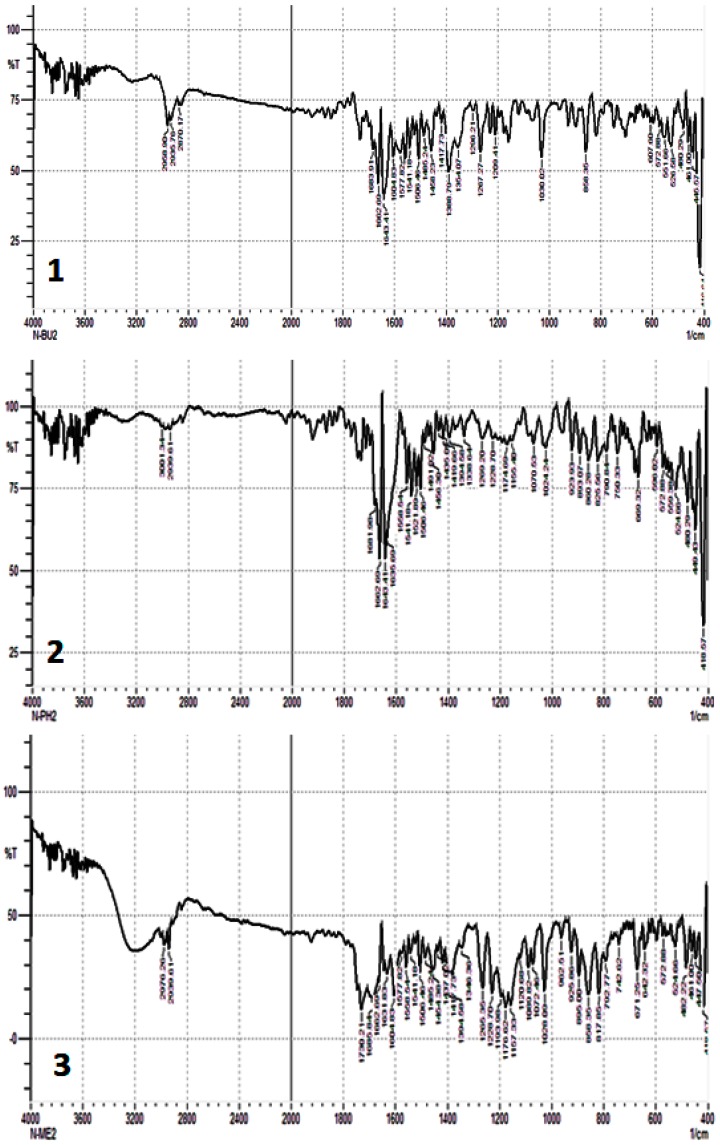
FT-IR spectra of Sn(IV) complexes **1**–**3**.

The absorption of C–Sn bond in the FT-IR spectra is highly dependent on the number and the nature of the substituents attached to the tin central atom. Selected FT-IR spectral data for Sn(IV) complexes **1**–**3** are reported in Table 2. In the FT-IR spectra of Sn(IV) complexes **1**–**3** (Figure 2), the bands in the 1651–1541 cm^−1^ region can be accounted for by the symmetric and asymmetric modes of the carboxylate carbonyl group (COO) [42]. The strong absorption peaks in the 1643–1651 cm^−1^ region are due to the asymmetric stretching vibration of COO, which is in agreement with the literature [43]. Furthermore, the COO asymmetric stretching vibration appeared within the 1541–1548 cm^−1^ region. The interaction between the tin atom and carboxylate oxygen can be established from the FT-IR stretching frequency, Δυ (COO) [44]. The Δυ [*v*_as_ (COO) − vs. (COO)] values of **1**–**3** were found to be in the range of 102 cm^−1^, which indicates a bidentate mode of interaction between the Sn and oxygen atoms [33], as a Δυ value of less than 200 cm^−1^ is commonly observed for bidentate interactions [33]. The FT-IR spectra of **1**–**3** also show new absorption bands in the range of 524–526 and 445–449 cm^−1^ regions, corresponding to the vibrations of Sn–C and Sn–O, respectively [45]. The observation of Sn–C and Sn–O peaks is an indication that coordination has occurred between Sn(IV) and the oxygen of the carboxylate group [46,47]. Based on the FT-IR spectral data, an octahedral geometry was proposed for the synthesized Sn(IV) complexes.

The NMR chemical shifts are highly dependent on the geometry of Sn(IV) complexes [48,49]. The chemical shifts are expected to be affected by coordination between the ligand and the tin atom via an effect known as a metal nuclear shielding [50]. The ^1^H-NMR spectra of **1**–**3** show characteristic doublets in the 1.42–1.47 ppm region owing to the methyl protons attached to the CH protons. In addition, the spectra exhibited quartet signals (3.87–3.77 ppm) corresponding to the CH protons. Moreover, the methoxy protons appeared as singlets at ca. 3.93 ppm. The ^1^H-NMR spectral data of **1**–**3** are reported in Table 3.

The ^13^C-NMR spectra of **1**–**3** show that the carbonyl carbon and the aromatic carbon attached to the methoxy group appeared at very low field (174.8–175.9 and 157.5–157.7 ppm, respectively). The CH carbons appear within the 43.7–45.0 ppm region. All other carbons appeared at the expected chemical shifts region [47]. The ^13^C-NMR spectral data of **1**–**3** are reported in Table 4.

**Figure 3:**
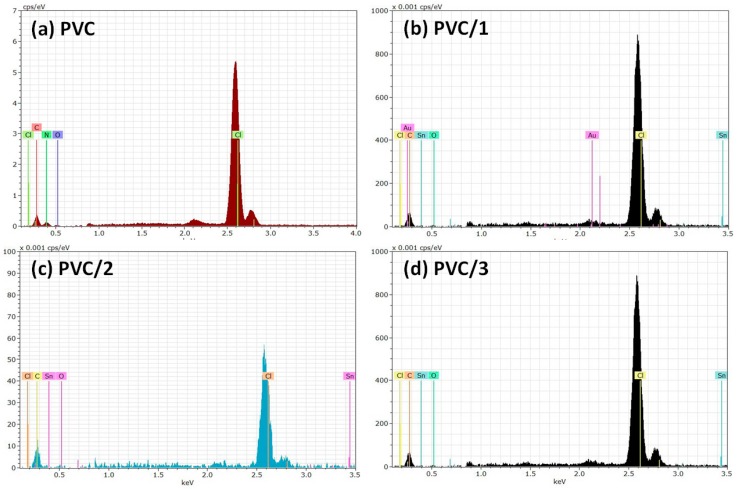
**Figure 3**. Energy-dispersive X-ray spectroscopy (EDX) spectra of the (**a**) blank poly(vinyl chloride) (PVC) film and the PVC films containing 0.5 wt % (**b**) complex **1**, (**c**) complex **2**, and (**d**) complex **3**.

Previous reports have indicated that the most desirable photostabilization effect is obtained when additives are added to PVC at a concentration of 0.5 wt % [32,33]. Therefore, Sn(IV) complexes **1**–**3** (0.5 wt %) were mixed with PVC and the corresponding polymeric films (with a thickness of 40 μm) were produced. Energy-dispersive X-ray spectroscopy (EDX) can be used to obtain information about the elemental composition of the polymeric films. The EDX spectra of the PVC films (Figure 3) show strong absorption peaks corresponding to the chlorine atom of PVC. The EDX spectra of the PVC containing complexes **1**–**3** also show a new band that is related to the tin atoms of complexes **1**–**3**. The assignment of the Sn peak is in agreement with previous reports [51].

### 2.2. Weight Loss of PVC Films

The weight loss of PVC during an irradiation process can be used as a measure of the level of photodegradation. Upon irradiation, a dehydrochlorination process occurs, resulting in the elimination of HCl from the PVC chains and the formation of polyene residues [52]. The PVC films containing complexes **1**–**3** were irradiated with a UV light for up to 300 h, and the PVC weight loss (%) was calculated using Equation (1), where *W*_1_ is the PVC weight before irradiation and *W*_2_ is the PVC weight after irradiation. The color of the PVC (blank) film and those containing Sn(IV) complexes **1**–**3** were almost identical before and after irradiation. The PVC films were colorless and darkened upon irradiation. After long term (300 h) of irradiation, the PVC films turned brown and less transparent,

(1) $$Weight\;loss\;\%=\left[\left(W_{1}-W_{2}\right)/W_{1}\right]\times 100.$$

**Figure 4:**
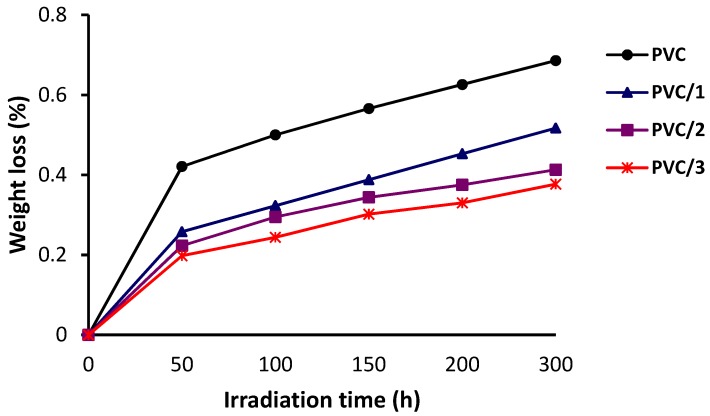
Effect of irradiation on the weight of PVC films.

The effect of irradiation time on PVC weight loss is shown in Figure 4. The PVC weight loss in the presence of **1**–**3** is reduced significantly compared with that in the blank PVC film. All the diorganotin(IV) complexes reduced the photodegradation of PVC significantly, but complex **3** was more effective than complexes **1** and **2**. For complex **3**, the weight loss over 300 h was less than 0.4%, whereas, for the blank PVC film, it was 0.7%.

### 2.3. FT-IR Spectroscopy of PVC Films

UV radiation alters the physical and chemical properties of polymers [53]. Photo-oxidation of PVC produces small fragments containing hydroxyl, carbonyl, and polyene moieties [53]. The intensities of the peaks corresponding to the hydroxyl (3500 cm^−1^), carbonyl (1722 cm^−1^), and polyene (1602 cm^−1^) groups in the FT-IR spectra of the PVC films can be monitored upon irradiation and compared with the intensity of a reference peak (1328 cm^−1^), which corresponds to the C–H bonds within the PVC chains. Such a comparison provides useful information about the rate of PVC photodegradation [54]. Therefore, the PVC films were irradiated, and the FT-IR spectra were recorded every 50 h up to 300 h. The index of each functional group (*I*_S_) was calculated from the absorbance of the functional group (*A*_s_) and the reference group (*A*_r_) using Equation (2),

(2) $$I_{s}=A_{s}/A_{r}.$$

**Figure 5:**
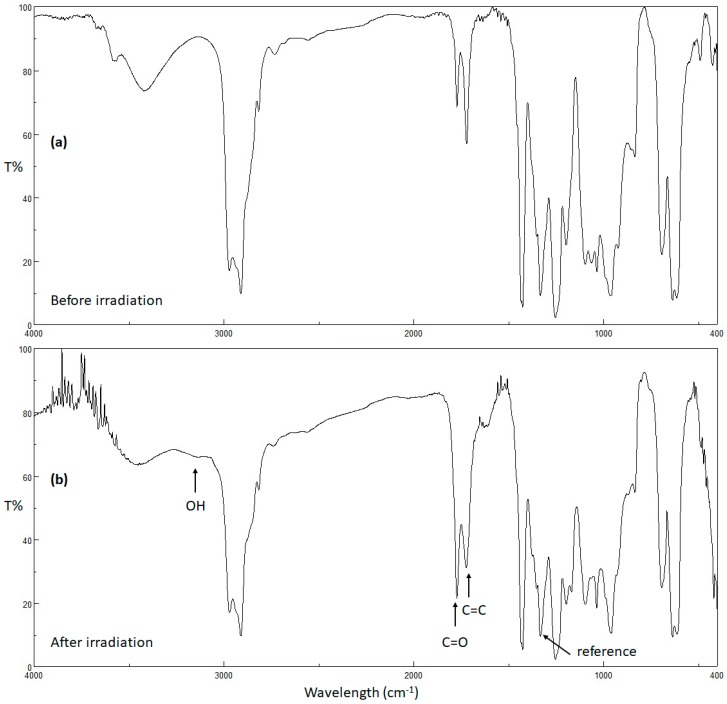
FT-IR spectra of PVC (blank) (**a**) before and (**b**) after irradiation.

**Figure 6:**
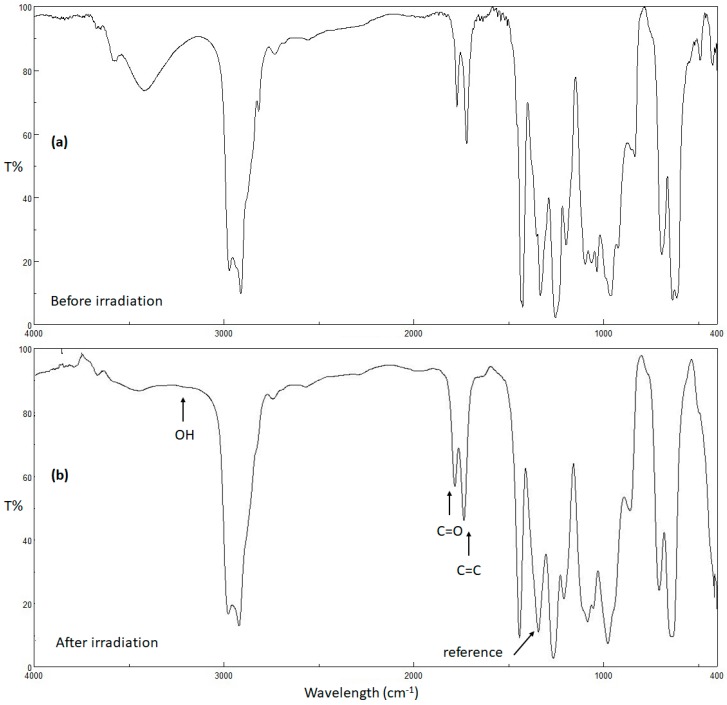
FT-IR spectra of PVC/**3** blend (**a**) before and (**b**) after irradiation.

**Figure 7:**
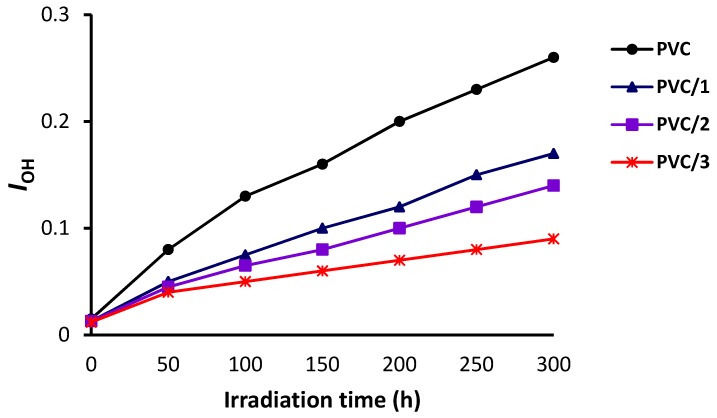
Effect of irradiation on the hydroxyl group index (*I*_OH_) of the PVC films.

**Figure 8:**
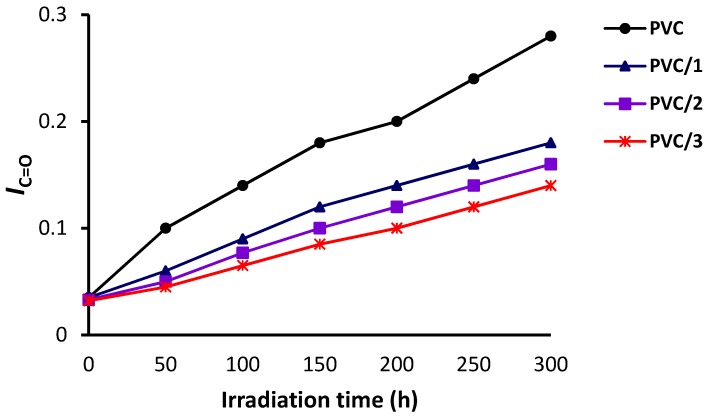
Effect of irradiation on the carbonyl group index (*I*_C=O_) of the PVC films.

**Figure 9:**
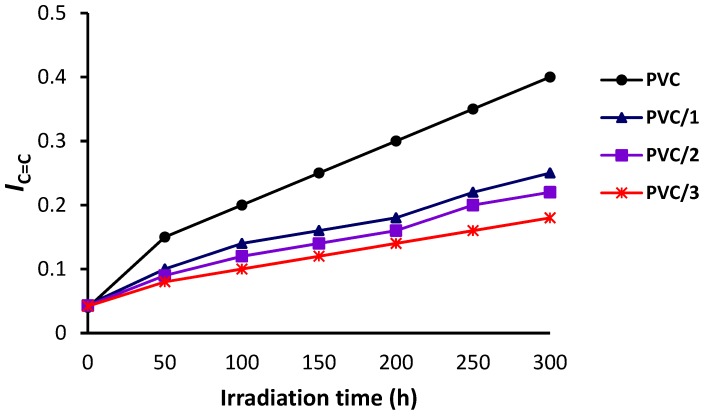
Effect of irradiation on the polyene index (*I*_C=C_) of the PVC films.

The changes in the FT-IR spectra of PVC (blank) film and PVC/**3** blend upon irradiation (300 h) are shown in Figure 5 and Figure 6, respectively. The indices for the carbonyl (*I*_C=O_), polyene (*I*_C=C_), and hydroxyl (*I*_OH_) groups were calculated for each PVC film at 50 h intervals and plotted *versus* irradiation time. As shown in Figure 7, Figure 8 and Figure 9, these indices were much smaller for the PVC films containing Sn(IV) complexes **1**–**3** than for the blank film. Evidently, complexes **1**–**3**, and in particular **3**, inhibit the photodegradation of PVC significantly. For example, *I*_C=O_, *I*_C=C_, and *I*_OH_ for the PVC film containing **3** after irradiation for 300 h were 0.09, 0.14, and 0.18, respectively, whereas those of the blank PVC film were 0.26, 0.28, and 0.40, respectively.

### 2.4. Molecular Weight of PVC Films

The intrinsic viscosity ([η]) of a polymeric solution can be used as a simple tool to determine the PVC average molecular weight (${\bar{M}}_{V}$). The photodegradation of PVC films leads to branching and cross-linking of the polymeric chains following the elimination of HCl, and, therefore, a decrease in ${\bar{M}}_{V}$ [55]. The PVC ${\bar{M}}_{V}$ can be calculated using Equation (3) based on constants *α* and *K* [56],

(3) $$\left[\eta \right]=K{\bar{M}}_{V}^{\alpha }.$$

**Figure 10:**
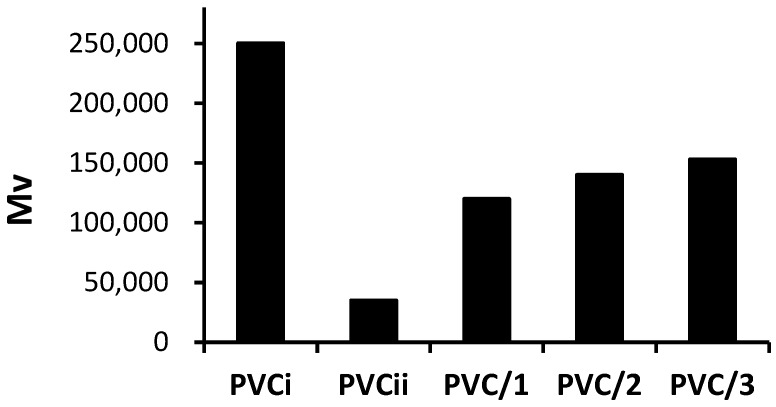
Effect of irradiation on the average molecular weight (${\bar{M}}_{V}$) of PVC films. PVCi and PVCii correspond to the ${\bar{M}}_{V}$ values of the blank PVC film before and after irradiation, respectively.

After irradiation for 300 h, the PVC films were dissolved in tetrahydrofuran, and the ${\bar{M}}_{V}$ values were calculated in the presence and absence of each Sn(IV) complex. The effect of irradiation (300 h) on the ${\bar{M}}_{V}$ values is shown in Figure 10. It is evident that the decrease in ${\bar{M}}_{V}$ was much greater for the blank PVC film than for the films containing the Sn(IV) complexes. After irradiation for 300 h, the ${\bar{M}}_{V}$ value of the blank PVC film decreases to 35,000 from 250,000 before irradiation. Irradiation had a much smaller effect on ${\bar{M}}_{V}$ in the PVC films containing the Sn(IV) complexes. For example, the ${\bar{M}}_{V}$ value of the PVC film containing complex **3** only decreased to 153,000 after irradiation for 300 h.

### 2.5. Surface Morphology of PVC Films

**Figure 11:**
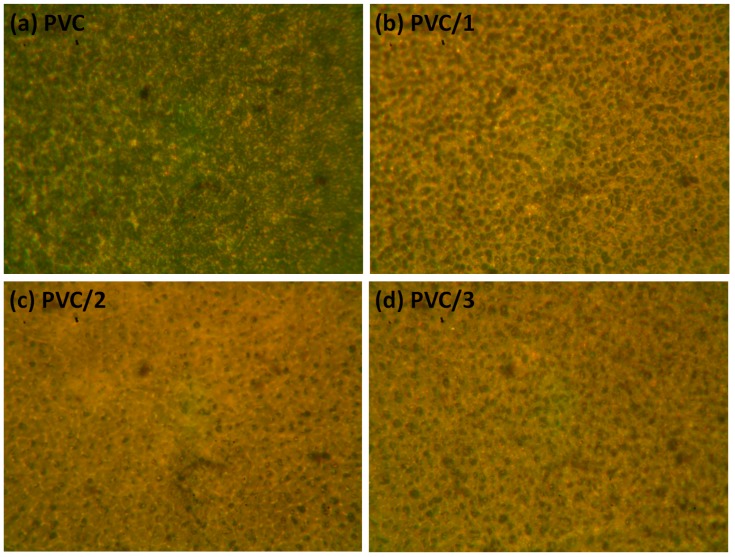
Surface morphologies (400× magnification) of (**a**) the blank PVC film and the PVC films containing (**b**) complex **1**, (**c**) complex **2**, and (**d**) complex **3** after irradiation for 300 h.

Optical microscopy provides evidence about the roughness and irregularity of the surface of a polymer film. In addition, it reveals defects, cracks, damage, decomposition, chain scission, and other changes that might occur within the polymer surface when irradiated with a UV light [20]. Such undesirable changes can be attributed to the dehydrochlorination process [57]. The surface morphology images (400× magnification) of the PVC films (Figure 11) after 300 h of continuous irradiation showed discoloration, rough surfaces, grooves, cracks, and white spots within the surface. However, the surface irregularities within the PVC films containing Sn(IV) complexes **1**–**3** were less noticeable than those appearing within the surface of the blank PVC film. This observation suggests that these complexes can reduce the rate of the dehydrochlorination process and therefore enhance the photostability of the irradiated PVC films.

### 2.6. Scanning Electron Microscopy (SEM) of PVC Films

**Figure 12:**
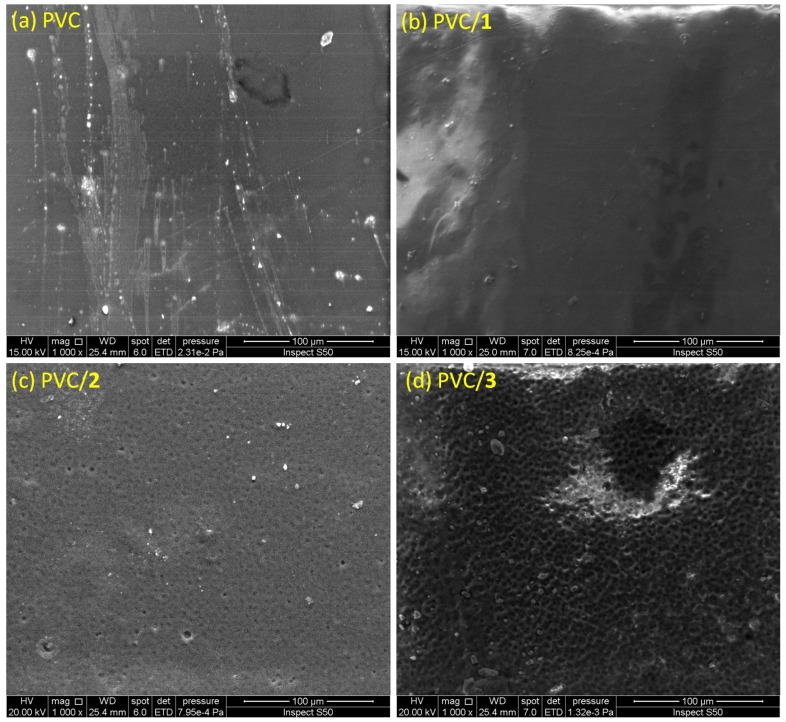
Scanning electron microscopy (SEM) images of (**a**) the blank PVC film and the PVC films containing (**b**) complex **1**, (**c**) complex **2**, and (**d**) complex **3** after irradiation for 300 h.

SEM images can be used to observe the changes caused by UV irradiation within the surface of the PVC films. In addition, they provide information about the particle size and shape, ionic conductivity, and thermal and mechanical stability of the polymeric matrix. Several reports have shown that SEM images of PVC surface before irradiation are neat, smooth, and homogeneous [33,35,36]. The SEM images of the surface of the blank PVC film and those containing Sn(IV) complexes **1**–**3** after 300 h of irradiation are shown in Figure 12. The SEM images show the formation of cavities within the PVC as a result of the photodegradation process. The lengths and depths of the cavities were larger in the blank PVC film than in the films containing the additives. The damage that occurs within the PVC surface is mainly a result of chain cross-linking following the elimination of HCl and other volatile degradation products from the polymeric chains [58].

**Figure 13:**
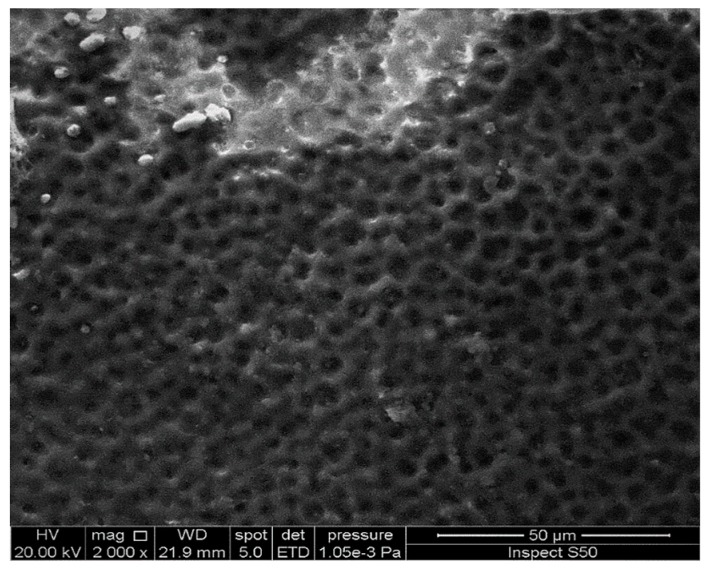
High-resolution SEM image of the PVC film containing complex **3** film after irradiation for 300 h.

The SEM image of the surface of the PVC film containing complex **3** shows more regular particle aggregation with hexagonal pores in a honeycomb like-structure (Figure 13). A similar observation has been made previously [35]. Clearly, complex **3** aids in the formation of a regular pore structure, likely because of slower dehydrochlorination and chain cross-linking processes. This phenomenon is possibly due to a strong interaction or coordination between the PVC chains and the Sn(IV) atom.

### 2.7. Atomic Force Microscopy (AFM) of PVC Films

**Figure 14:**
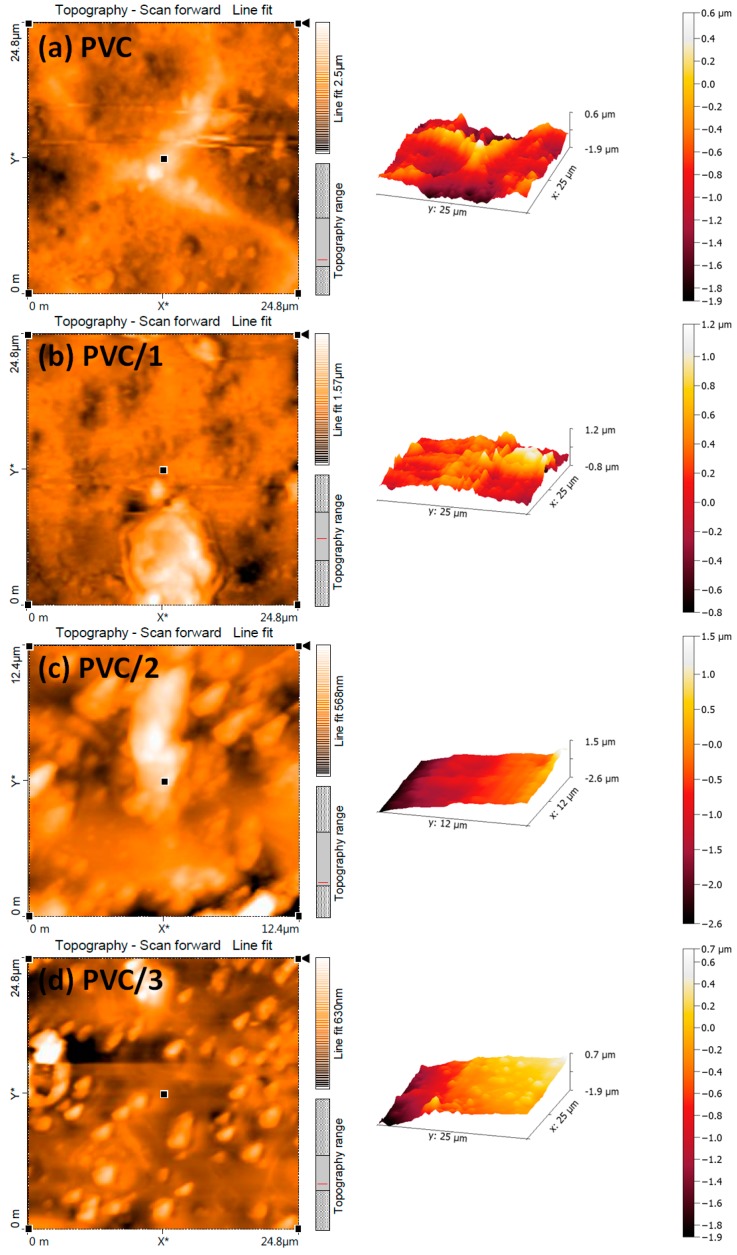
2D and 3D atomic force microscopy (AFM) images of (**a**) the blank PVC film and the PVC films containing (**b**) complex **1**, (**c**) complex **2**, and (**d**) complex **3** after irradiation for 300 h.

AFM provides information about the surface roughness and pore sizes of polymers [59]. Previous reports have shown that nonirradiated PVC films have smooth surfaces that contain a limited number of holes [20,37]. After irradiation, the 2D and 3D AFM images showed that the PVC films containing the Sn(IV) complexes (Figure 14) had much smoother surfaces with fewer holes than the blank PVC film. Indeed, after irradiation, the roughness factor (*R*q) was much higher for the blank PVC film than for the films containing the Sn(IV) complexes (Table 5). The roughness factor measures the changes in physical properties, due to either cleavage of the C–C or C–Cl bonds or photo-oxidation process within the polymeric chains, which is minimal in the case of PVC/**3** blend.

### 2.8. Photostabilization Mechanism

**Figure 15:**
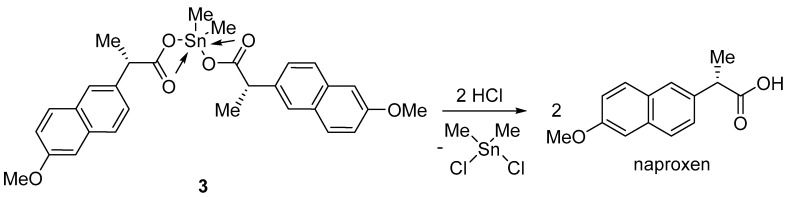
Diorganotin(IV) complex **3** acting as an HCl scavenger.

Several mechanisms can be suggested to explain the role played by the diorganotin(IV) complexes as photostabilizers against the photodegradation of PVC films. Sn(IV) is a strong Lewis acid and acts as an efficient HCl scavenger. As photoirradiation of PVC leads to the elimination of HCl through a dehydrochlorination process, the tin atoms in complexes **1**–**3** could capture the chloride ions, leading to the formation of naproxen and Me_2_SnCl_2_. This process would eliminate the harmful effects of HCl on the polymeric chains (Figure 15). Thus, diorganotin(IV) complexes **1**–**3** could induce long-term protection of PVC against photodegradation by acting as secondary photostabilizers [28].

**Figure 16:**
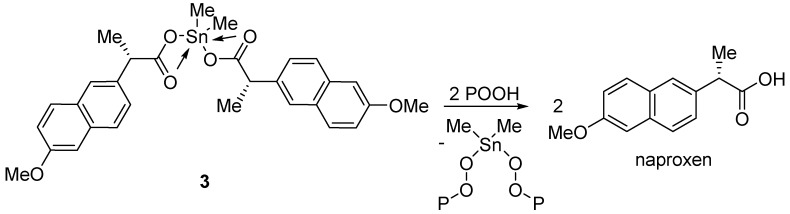
Diorganotin(IV) complex 3 acting as a peroxide decomposer.

Diorganotin(IV) complexes **1**–**3** could also stabilize PVC against photodegradation by acting as peroxide decomposers. In the presence of oxygen, the photodegradation of PVC produces radicals, which on reaction with oxygen lead to the formation of peroxide radicals [60]. Complexes **1**–**3** can react with peroxides, such as hydroperoxides, and, therefore, enhance the photostability of polymeric films upon photo-oxidation (Figure 16) [61].

The photostabilization of PVC in the presence of diorganotin(IV) complexes **1**–**3** could also be due to the formation of coordination bonds between the polarized oxygen atoms of the carboxylate groups within the naproxen moieties and the polarized carbon atoms of the C–Cl bonds within the polymeric chains. Complexes **1**–**3** could absorb light energy directly (e.g., act as primary photostabilizers) and then eliminate such energy at a harmless rate over time [62]. Clearly, diorganotin(IV) complex **3** was the most effective photostabilizer among those examined in this study. This complex contains a small methyl substituent that has no steric hindrance compared with butyl and phenyl substituents of the other complexes. The steric hindrance of these groups could reduce the efficiency of complexes **1** and **2** as primary stabilizers.

## 3. Materials and Methods

### 3.1. General

Chemicals and reagents were purchased from Sigma-Aldrich (Schnelldorf, Germany). PVC (${\bar{M}}_{V}$ = ca. 250,000, polymerization degree = 800, K value = 67) was purchased from Petkim Petrokimya (Istanbul, Turkey). FT-IR spectra were recorded on a Shimadzu FTIR 8300 spectrophotometer (Kyoto, Japan) in the spectral range of 400–4000 cm^−1^ using the KBr disc technique. Elemental analyses were performed using a Vario EL III instrument (Analysensysteme GmbH, Hanau, Germany). The melting points were recorded on a Mitamura Riken Kogyo MPD melting point apparatus (Tokushima, Japan). ^1^H- (300 MHz) and ^13^C-NMR (75 MHz) spectra were recorded on a Bruker DRX300 NMR spectrometer (Bruker, Zurich, Switzerland) in DMSO-*d*_6_. The EDX measurements were carried out on Bruker XFlash^®^ 6|10 detector (Tokyo, Japan). The optical images of the PVC surface were obtained using a Meiji Techno Microscope (Tokyo, Japan). The SEM images were recorded on a TESCAN FE-SEM MIRA3 system (Kohoutovice, Czech Republic). The AFM images were recorded on a Veeco instrument (Plainview, NY, USA). An accelerated weather-meter QUV tester (Q-Panel Company, Homestead, FL, USA) was used for irradiation of the films with a UV light (λ_max_ at 365 nm at a light intensity of 6.43 × 10^–9^ ein dm^–3^ s^−1^) at room temperature. A Digital Caliper DIN 862 micrometer (Vogel GmbH, Kevelaer, Germany) was used to determine the thickness of the PVC films (40 µm). The PVC films were fixed using aluminum plate stands (Q-Panel Company, Homestead, FL, USA).

### 3.2. Synthesis of Sn(IV) Complexes ***1**–**3***

A solution of naproxen (0.46 g, 2.0 mmol) in MeOH (30 mL) was slowly added to a stirred solution of the appropriate dialkyltin chloride (1.0 mmol) in MeOH. The mixture was refluxed for 8 h, and then the solvent was removed under reduced pressure. The obtained solid obtained was recrystallized from MeOH to give the corresponding Sn(IV) complex (**1**–**3**).

### 3.3. Preparation of PVC Films

Diorganotin(IV) complexes **1**–**3** (25 mg) were added to a stirred solution of PVC (5.0 g) in tetrahydrofuran (100 mL). The homogenous mixture was stirred at room temperature for 30 min and then poured into glass plates containing 15 holes (ca. 40 μm). The solvent was allowed to evaporate at room temperature for 24 h and then under vacuum for 24 h to obtain the PVC films.

## 4. Conclusions

New highly aromatic diorganotin(IV) complexes were synthesized, characterized, and evaluated as photostabilizers for PVC during long-term UV irradiation. The degradation of PVC was reduced significantly in the presence of a low concentration (0.5 wt %) of the diorganotin(IV) complexes. In addition, the surfaces of the polymeric materials containing additives were much smoother than that of the blank PVC film after irradiation with UV light. The diorganotin(IV) complex that contained the smallest substituent (methyl group) was found to be the most effective additive for stabilizing the PVC film. SEM images showed that the polymeric film containing this complex formed a honeycomb-like structure with regularly distributed hexagonal pores. The diorganotin(IV) complexes could inhibit PVC degradation by acting as direct absorbers of the UV irradiation, by acting as radical or HCl scavengers, or by interacting with the polymeric chains through the formation of coordination bonds. For the potential application of the synthesized naproxen diorganotin(IV) complexes as PVC photostabilizers, the hazard associated with the possible leakage of naproxen and tin needs to be investigated.

## Figures and Tables

**Table 1:** Physical properties and elemental analysis of Sn(IV) complexes **1**–**3**.

Sn(IV) Complex	R	Color	Yield (%)	Melting Point (°C)	Calcd. (Found) (%)	
					C	H
**1**	Bu	white	79	106–108	62.53 (62.58)	6.41 (6.57)
**2**	Ph	brown	85	82–84	65.68 (65.42)	4.96 (5.12)
**3**	Me	pale orange	86	129–131	59.33 (59.57)	5.31 (5.49)

**Table 2:** Selected FT-IR spectral data for Sn(IV) complexes **1**–**3**.

Sn(IV) Complex	FT-IR (*ν*, cm^−1^)				
	C=O (as)	C=O (s)	C=C	Sn–C	Sn–O
**1**	1643	1541	1458	526	445
**2**	1643	1541	1456	524	449
**3**	1651	1548	1454	524	447

**Table 3:** ^1^H-NMR spectral data for Sn(IV) complexes **1**–**3**.

Sn(IV) Complex	^1^H-NMR (300 MHz; DMSO-*d*_6_, ppm)
**1**	7.78–7.71 (m, 6H, Ar), 7.35 (d, *J* = 7.8 Hz, 2H, Ar), 7.27 (s, 2H, Ar), 7.13 (d, *J* = 7.8 Hz, 2H, Ar), 3.93 (s, 6H, 2OMe), 3.77 (q, *J* = 7.1 Hz, 2H, 2CH), 1.42 (d, *J* = 7.1 Hz, 6H, 2Me), 1.22–1.19 (m, 8H, 4CH_2_), 1.00 (m, 4H, 2CH_2_), 0.57 (t, *J* = 7.4 Hz, 6H, 2Me)
**2**	7.82–7.46 (m, 16H, Ar), 7.40 (d, *J* = 7.6 Hz, 2H, Ar), 7.29 (s, 2H, Ar), 7.15 (d, *J* = 7.6 Hz, 2H, Ar), 3.94 (s, 6H, 2OMe), 3.87 (q, *J* = 7.2 Hz, 2H, 2CH), 1.47 (d, *J* = 7.2 Hz, 6H, 2Me)
**3**	7.81–7.71 (m, 6H, Ar), 7.39 (d, *J* = 7.6 Hz, 2H, Ar), 7.28 (s, 2H, Ar), 7.14 (d, *J* = 7.6 Hz, 2H, Ar), 3.93 (s, 6H, 2OMe), 3.83 (q, *J* = 7.2 Hz, 2H, 2CH), 1.42 (d, *J* = 7.2 Hz, 6H, 2Me), 0.57 (s, 6H, 2Me)

**Table 4:** ^13^C-NMR spectral data for Sn(IV) complexes **1**–**3**.

Sn(IV) Complex	^13^C-NMR (75 MHz; DMSO-*d*_6_, ppm)
**1**	175.9 (C=O), 157.5, 133.7, 133.1, 129.5, 129.1, 128.0, 127.5, 125.9, 119.0, 106.2, 55.6 (OMe), 43.7 (CH), 31.5 (CH_2_), 26.0 (CH_2_), 25.6 (CH_2_), 15.9 (Me), 15.0 (Me)
**2**	175.8 (C=O), 157.7, 136.7, 136.2, 133.8, 133.6, 129.6, 129.4, 128.9, 128.7, 128.0, 127.8, 126.9, 119.1, 106.2, 55.6 (OMe), 44.7 (CH), 18.9 (Me)
**3**	174.8 (C=O), 157.6, 136.8, 133.7, 129.6, 128.9, 127.5, 126.9, 126.0, 119.3, 106.2, 55.6 (OMe), 45.0 (CH), 18.9 (Me), 18.5 (Me)

**Table 5:** Roughness factor (*R*q) for PVC after irradiation.

Photoirradiated PVC Film (300 h)	*R*q
PVC blank	457.6
PVC/**1**	130.3
PVC/**2**	126.6
PVC/**3**	87.5
